# The loss of nuclear expression of single-stranded DNA binding protein 2 of gastric adenocarcinoma and its prognostic role: Analysis of molecular subtype

**DOI:** 10.1371/journal.pone.0236896

**Published:** 2020-08-03

**Authors:** Seongsik Bang, Hyunsung Kim, Kiseok Jang, Seung Sam Paik, Su-Jin Shin

**Affiliations:** 1 Departments of Pathology, College of Medicine, Hanyang University, Seoul, Republic of Korea; 2 Department of Pathology, Gangnam Severance Hospital, Yonsei University College of Medicine, Seoul, Republic of Korea; Istituto di Ricovero e Cura a Carattere Scientifico Centro di Riferimento Oncologico della Basilicata, ITALY

## Abstract

Single-stranded DNA binding protein 2 (SSBP2) is ubiquitously expressed, with several studies reporting it to be a tumor suppressor. We investigated SSBP2 expression and its clinicopathological significance in gastric cancer. SSBP2 expression was examined by immunohistochemistry in 539 gastric cancer sections. The cases were divided into three subtypes, namely, Epstein–Barr virus-associated (EBV), microsatellite unstable, and others (microsatellite stable and EBV negative), based on the molecular classification of The Cancer Genome Atlas (TCGA). Cases were also divided into two subgroups according to the amplification status of human epidermal growth factor receptor 2 (HER2). Most cases showed SSBP2 positivity, and only 24 (4.5%) cases displayed negative nuclear expression. Loss of nuclear expression correlated significantly with high pT category (*P* = 0.001), nodal metastasis (*P* = 0.002), and stage of progression (*P* = 0.005), with no correlation between molecular characteristics and SSBP2 expression. All HER2 amplification cases displayed positive SSBP2 expression. Negative SSBP2 cases showed significantly shorter recurrence-free survival (RFS) compared to positive SSBP2 cases (*P* = 0.008). Loss of nuclear expression of SSBP2 was significantly associated with shorter RFS in the microsatellite stable and EBV negative groups (*P* = 0.002), as well as the HER2 negative group (*P* = 0.007). However, there were no statistically significant differences in multivariate analyses. Loss of nuclear expression of SSBP2 was a poor prognostic factor, associated with stage of progression and recurrence, and showed no significant difference in molecular characteristics, including TCGA subtype and HER2 status.

## Introduction

The GLOBOCAN database (September 2018 edition) of The International Agency for Research on Cancer (IARC) indicates that gastric cancer is the sixth most common cancer and the third most common cause of mortality worldwide, with the highest incidence being in Eastern Asia, including Korea [1, 2].

Gastric cancer is a common malignant tumor of the digestive system and a heterogeneous disease with diverse histopathological characteristics. Therefore, several histological classifications such as the Lauren classification (intestinal, diffuse, mixed and indeterminate type) and WHO classification (tubular, papillary, mucinous, and poorly cohesive carcinoma) are available [3, 4]. The Cancer Genome Atlas (TCGA) research network recently divided the molecular classification of gastric cancer into four subgroups: Epstein–Barr virus (EBV), microsatellite instability (MSI), genomic stability (GS), and chromosomal instability (CIN) associated tumors [5]. Surgical resection and adjuvant therapy are the main treatment modalities. In advanced gastric cancers (AGC), the probability of metastasis or peritoneal seeding dissemination is still high with poor overall prognosis [6]. Many studies have therefore been conducted on molecular targeted therapies in addition to conventional chemotherapy [7].

Single-stranded DNA binding protein 2 (SSBP2) is known to be a candidate tumor suppressor in patients with myeloid leukemia located at chromosome 5q14 [8–10]. SSBP2 binds to the transcriptional cofactor Lim-domain-binding protein 1 (LDB1) and enhances LDB1 stability to regulate gene expression [11]. The role of SSBP2 has also been studied in several solid tumors including hepatocellular carcinoma, gallbladder cancer, esophageal squamous cell carcinoma, and prostate cancer. Most studies, except for a recent report on hepatocellular carcinoma, have reported SSBP2 to have tumor suppressive action [12–15]. Maldonado *et al*. [16] found that decreased SSBP2 expression was associated with an increased risk of recurrence in advanced prostate cancer. However, no studies are available regarding the expression of SSBP2 in gastric cancer.

Therefore, we tried to identify the expression of SSBP2 through immunohistochemistry in gastric cancer and analyzed the association of expression and prognosis. The differences in SSBP2 expression, according to molecular subtypes and HER2 status, were also investigated.

## Materials and methods

### Patients

We retrospectively collected 561 gastric cancer patients who underwent surgical resection at Hanyang University Hospital, between February 2005 and August 2010. Among them, 505 patients underwent gastrectomy, 54 patients had received endoscopic submucosal dissections (ESD), and two patients had received wedge resections. Two patients with AGC were excluded from the study due to neoadjuvant chemotherapy. We also excluded patients who had distant metastasis at the time of AGC diagnosis (seven cases), or who did not have an adequate surgical margin (one case). Twelve cases were excluded as they did not have enough tumor tissue for the study (AGC cases: two, early gastric cancer cases undergoing gastrectomy: three, early gastric cancer cases undergoing ESD: seven). Consequently, 539 patients were included in this study. Medical records were reviewed to determine the following clinical characteristics: age, sex, follow-up interval, and survival, and recurrence status.

### Ethics statement

Data anonymization was performed after enrolling 561 patients and completing a case report form. We deleted all personally identifiable information and constructed a database that can distinguish each case only by case number. The study protocol conformed to the ethical guidelines of the 1975 Declaration of Helsinki and was approved by the Institutional Review Board of the Hanyang University Hospital (HYUH 2019-04-032-001), and the requirement for informed consent was waived.

### Pathological evaluation

We reviewed all hematoxylin and eosin (H&E) slides used at the time of diagnosis. Pathologic features included tumor location (center of tumor), tumor size, gross type, histological type of WHO classification, Lauren classification, lymphatic or vascular invasion, perineural invasion, and stage of tumor-node-metastasis (TMN). Cardia, fundus, and body were considered for the proximal stomach, while angle, antrum, and pylorus were regarded for the distal stomach. Early gastric cancer (EGC) was classified as type I to type III. Type II tumors were further divided into IIa, IIb, and IIc. When two or more gross types coexisted, they were classified as mixed type. The gross appearance of AGC was classified as type 1 to type 4, according to Borrmann’s classification [3]. It was regarded as a lymphatic invasion when the tumor invaded the small vessel. The invasion of the large vessel with the muscle layer was regarded as a venous invasion. The histological type was based on 2010 WHO classifications, with TMN stage based on the American Joint Committee on Cancer (AJCC) 8th edition. Pathological evaluations were performed by two pathologists, independently (SSB and SJS).

### Tissue microarray construction

Tissue microarray (TMA) was prepared using a tissue microarray system (Tissue Microarray Set, Labro, Seoul, Korea). Formalin-fixed paraffin-embedded (FFPE) tissue samples taken from resected primary tumors at the time of initial diagnosis were collected exclusively. The representative tumor area was selected by light microscopy of H&E-stained sections, and 3.0 mm core tissue microarray (TMA) blocks were constructed with two representative cores for each case.

### Immunohistochemistry and interpretation

The immunohistochemical staining for SSBP2 was performed in 4 μm sections obtained from TMA blocks. Deparaffinization was performed by immersing the sections in xylene solution. Rehydration was performed using a graded series of ethanol. Heat-induced epitope retrieval was performed by autoclave heating at 100 °C for 20 min in sodium citrate buffer (pH 6.0). After cooling, blocking of endogenous peroxidase was performed using a peroxidase-blocking solution (S2023, Dako, Glostrup, Denmark) for 15 min. The sections were incubated in a humidified chamber with a diluted (1:200) primary antibody overnight at 4°C. The recombinant anti-SSBP2 antibody (ab177944, Abcam, Cambridge, UK) was used as a primary antibody. The sections were subsequently incubated with a Labeled Polymer (K5007, EnVision^TM^ Detection System, Dako) for 30 min at room temperature. We used 3,3'-diaminobenzidine tetrahydrochloride (DAB) chromogen (K5007, EnVision^TM^ Detection System, Dako) to detect the antigen-antibody complex. Finally, hematoxylin (Mayer’s) counterstain and mounting coverslips were performed on the slides.

**Fig 1:**
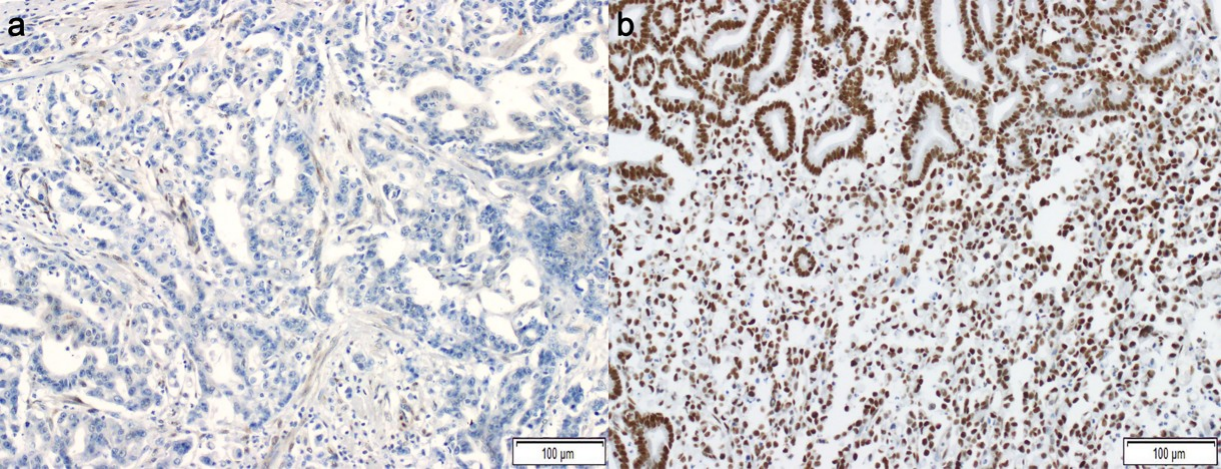
Immunohistochemical staining of single-stranded DNA binding protein 2 (SSBP2) in gastric adenocarcinoma (a: negative, b, positive; x200; scale bar: 100 μm).

SSBP2 expression was evaluated by two pathologists (SB and SS) who were blinded to clinical data, according to tumor cell nuclear staining using a light microscope. Tumor cells showing positive nuclear staining was defined as positive SSBP2 expression, regardless of intensity and proportion. Tumor cells showing a complete lack of nuclear staining were defined as negative (Fig 1).

### Microsatellite instability status

Immunohistochemical staining of MLH1 (G168-728, Cell Marque, CA, USA), PMS2 (MRQ-28, Cell Marque, CA, USA), MSH2 (G219-1129, Cell Marque, CA, USA), and MSH6 (PU29, Leica Biosystems, Nussloch, Germany) was performed to confirm the expression of mismatch repair proteins. Staining was performed in representative blocks (whole sections) of primary tumors. The cases showing loss of expression in all tumor cells were defined as negative. The nuclei of normal epithelium and inflammatory cells were regarded as internal controls. The tumors positive for all four immunohistochemical stains were classified as microsatellite stable (MSS). Those that gave negative results for one or more of the four immunohistochemical stains were classified as MSI.

### Epstein-Barr virus status

We detected EBV through the EBV-encoded RNA in situ hybridization (EBER-ISH) technique. The INFORM EBER Probe (Roche, Basel, Switzerland) was used to perform automated staining on TMA slides, according to the manufacturer's instructions. Positive staining was defined by diffuse staining of tumor cells.

### HER2 status

HER2 DNA amplification was assessed with the silver DNA in situ hybridization (SISH) technique on TMA slides. The INFORM HER2 Dual ISH DNA probe cocktail (Roche, Basel, Switzerland), with HER2 probe and chromosome 17 (CEP17) probe, was used for staining. Automated staining was performed according to the protocol of Ventana Benchmark XT staining systems. The number of black (HER2) and red (CEP17) signals were counted. We considered that a HER2 / CEP17 ratio of ≥ 2.0 was amplified, and a HER2 / CEP17 ratio of <2.0 was not amplified.

### Statistical analysis

The correlation between SSBP2 expression and clinicopathologic features was calculated with Pearson's chi-square (χ2) or Fisher’s exact tests. Overall survival (OS) was defined as the time from diagnosis with gastric cancer to death. Recurrence-free survival (RFS) was defined as the time from surgical treatment to the first recurrence, either clinically or pathologically. Survival curves were constructed using the Kaplan–Meier method and the influence of variables was analyzed using a log-rank test. The univariate and multivariate Cox regression analyses were used to determine significant prognostic variables. In all analyses, two-sided P values <0.05 were regarded as statistically significant. Data analysis was performed using SPSS software version 25.0 (IBM, Armonk, USA).

## Results

### Clinicopathologic and molecular characteristics

The clinicopathologic characteristics of 539 patients are summarized in S1 Table. The median follow-up period was 118 months (range, 1–166 months). Among 539 patients, 302 (56.0%) patients received curative surgical resection only, and 237 (44.0%) patients received curative surgical resection plus adjuvant chemotherapy. Of 237 patients, 129 (23.9%) patients received fluoropyrimidine-based chemotherapy, 6 (1.1%) patients received platinum-based chemotherapy, and 102 (18.9%) patients received fluoropyrimidine plus platinum chemotherapy. According to the TCGA molecular subtypes of gastric adenocarcinoma, the 539 gastric cancer cases were classified into EBV (35 cases, 6.5%), MSI (44 cases, 8.2%), and ‘others’ (460 cases, 85.3%) subgroups. EBER-ISH positive cases were considered to be of EBV subtype, whereas, cases were classified as MSI when one or more of the four mismatch repair proteins displayed negative results. GS and CIN subtypes were not distinguished in this study but rather classified as EBV negative or MSS (S2 Table). HER2 status was classified separately from TCGA subtypes. HER2 amplification was seen in 26 cases (4.8%) and none of these were included in the EBV or MSI subtypes.

### SSBP2 expression and clinicopathological features

The correlation between SSBP2 expression and clinicopathological features in patients with gastric adenocarcinoma is shown in Table 1. Overall, 515 cases (95.5%) were positive for nuclear staining, and 24 cases (4.5%) displayed complete negative staining. Loss of nuclear SSBP2 expression was significantly correlated with high pT category (*P* = 0.001), nodal metastasis (*P* = 0.002), and stage of progression (*P* = 0.005). No statistically significant correlations were found between SSBP2 expression and other clinicopathological features.

**Table 1:** Correlation between single-stranded DNA binding protein 2 expression and clinicopathological features in patients with gastric adenocarcinoma (n = 539).

Variables	Negative (n = 24)	Positive (n = 515)	*P*-value
**Age**			0.314
< 65 years	17 (5.2%)	312 (94.8%)	
≥ 65 years	7 (3.3%)	203 (96.7%)	
**Sex**			0.581
Male	18 (4.8%)	359 (95.2%)	
Female	6 (3.7%)	156 (96.3%)	
**Location**			0.324
Proximal	11 (5.6%)	185 (94.4%)	
Distal	13 (3.8%)	330 (96.2%)	
Histologic type*			0.607
Differentiated	12 (5.0%)	230 (95.0%)	
Undifferentiated and others	12 (4.0%)	285 (96.0%)	
**Lauren**			0.378
Intestinal	14 (5.2%)	253 (94.8%)	
Diffuse and mixed	10 (3.7%)	262 (96.3%)	
**pT category**			0.001
pT1 and pT2	8 (2.3%)	336 (97.7%)	
pT3 and pT4	16 (8.2%)	179 (91.8%)	
**Nodal status**			0.002
Negative	7 (2.2%)	315 (97.8%)	
Positive	17 (7.8%)	200 (92.2%)	
**Stage**^†^			0.005
I	7 (2.3%)	300 (97.7%)	
II and III	17 (7.3%)	215 (92.7%)	
**Lymphovascular invasion**			0.079
Absent	8 (2.9%)	266 (97.1%)	
Present	16 (6.0%)	249 (94.0%)	
**Perineural invasion**			0.073
Absent	11 (3.2%)	329 (96.8%)	
Present	13 (6.5%)	186 (93.5%)	

*Differentiated: well-differentiated, moderately differentiated adenocarcinoma; undifferentiated: poorly differentiated, signet ring cell carcinoma; others: papillary, mucinous, adenosquamous, hepatoid, gastric carcinoma with lymphoid stroma, adenocarcinoma with choriocarcinomatous differentiation

^†^AJCC 8^th^ edition.

### SSBP2 expression and molecular characteristics

There was no correlation between molecular characteristics and SSBP2 expression. One EBV positive case and three MSI cases displayed a loss of nuclear SSBP2 expression. Of the 460 EBV negative and MSS cases (CIN or GS subtype), 20 cases (4.3%) showed a loss of SSBP2 expression. All 26 HER2 amplification cases showed positive SSBP2 expression (Table 2).

**Table 2:** Correlation between single-stranded DNA binding protein 2 expression and molecular characteristics in patients with gastric adenocarcinoma (n = 539).

Variables	Negative (n = 24)	Positive (n = 515)	*P*-value
**EBV status**			
Negative	23 (4.6%)	481 (95.4%)	1.000
Positive	1 (2.9%)	34 (97.1%)	
**MSI status**			
MSS	21 (4.2%)	474 (95.8%)	0.434
MSI	3 (6.8%)	41 (93.2%)	
**HER2 status**			
No amplification	24 (4.7%)	489 (95.3%)	0.622
Amplification	0 (0.0%)	26 (100%)	

Abbreviations: EBV, Epstein–Barr virus; MSI, Microsatellite instability; MSS, microsatellite stable.

### Prognostic significance of SSBP2 expression

**Fig 2:**
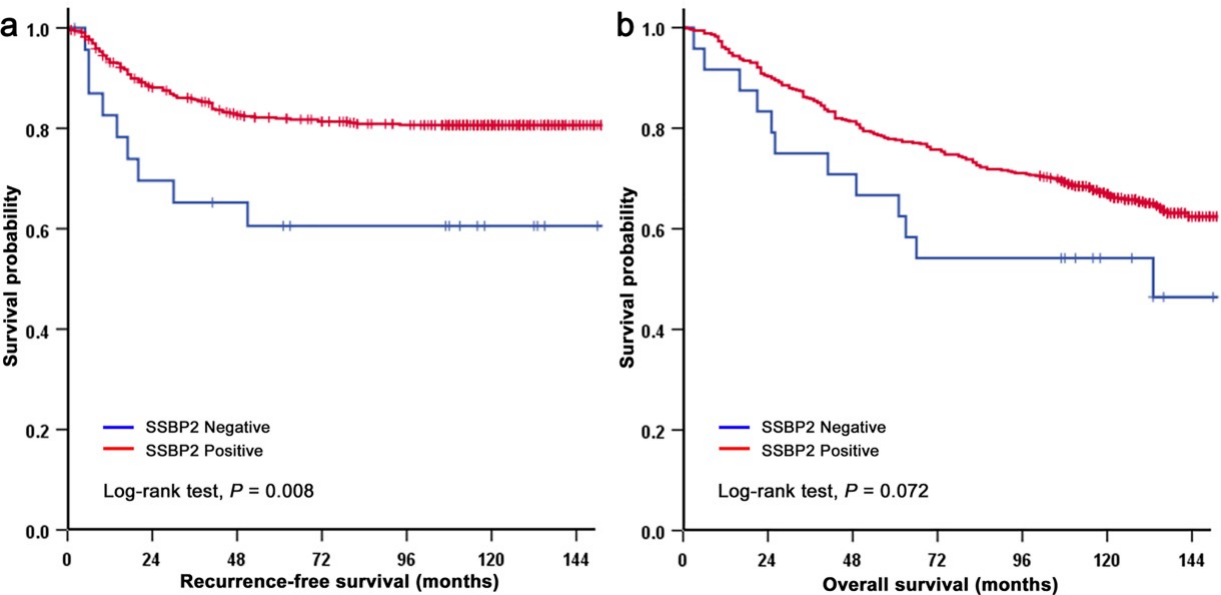
Kaplan-Meier analysis of SSBP2 in gastric adenocarcinoma. (a) Recurrence-free survival (RFS) was significantly worse in patients with loss of SSBP2 expression compared to those with positive expression, and (b) overall survival (OS) was worse in patients with loss of SSBP2 expression compared to those with positive expression.

In the 539 patients with gastric carcinomas, loss of SSBP2 expression was associated with shorter RFS and OS (*P* = 0.008 and *P* = 0.072, respectively; Fig 2). Univariate analyses revealed that other factors might be associated with a shorter RFS, including undifferentiated histological type (*P* = 0.001), diffuse and mixed type of Lauren classification (*P* < 0.001), high pT category (*P* < 0.001), nodal metastasis (*P* < 0.001), high AJCC stage (*P* < 0.001), lymphovascular invasion (*P* < 0.001), and perineural invasion (*P* < 0.001). In the multivariate analysis, a high AJCC stage (*P* < 0.001) was significantly related to poor prognostic factors, while a loss of SSBP2 expression was not statistically significant (Table 3).

**Table 3:** The univariate and multivariate Cox regression analyses for recurrence-free survival (RFS) and overall survival (OS) in patients with gastric adenocarcinoma (n = 539).

**Recurrence-free survival (RFS)**						
**Variables**	**Univariate analysis**			**Multivariate analysis**		
	**HR**	**95% CI**	***P-*values**	**HR**	**95% CI**	***P-*values**
**SSBP2 expression** (positive vs. negative)	2.438	1.231–4.828	0.008	1.646	0.828–3.273	0.155
**Age group** (<65 vs. ≥65)	1.019	0.687–1511	0.926			
**Sex** (female vs. male)	1.029	0.677–1.566	0.892			
**Location** (distal vs. proximal)	1.127	0.761–1668	0.551			
Histologic type^*^ (differentiated vs. undifferentiated and others)	2.040	1.341–3.102	0.001			
**Lauren classification** (intestinal vs. diffuse and mixed)	2.304	1.526–3.479	<0.001	1.452	0.955–2.208	0.081
**pT category** (T1-2 vs. T3-4)	11.175	6.720–18.583	<0.001			
**Nodal status** (negative vs. positive)	10.841	6.361–18.476	<0.001			
**Stage**^†^ (I vs. II, III)	14.857	7.950–27.768	<0.001	5.642	2.388–13.330	<0.001
**Lymphovascular invasion** (absent vs. present)	10.981	5.877–20.518	<0.001	2.177	0.948–4.998	0.066
**Perineural invasion** (absent vs. present)	8.307	5.187–13.303	<0.001	1.576	0.868–2.863	0.135
**EBV status** (negative vs. positive)	1.001	0.465–2.155	0.998			
**MSI status** (MSI vs. MSS)	23.217	1.234–436.886	0.036			
**HER2 amplification** (negative vs. positive)	1.247	0.547–2.843	0.599			
**Overall survival (OS)**						
**Variables**	**Univariate analysis**			**Multivariate analysis**		
	**HR**	**95% CI**	***P-*values**	**HR**	**95% CI**	***P-*values**
**SSBP2 expression** (positive vs. negative)	1.698	0.946–3.047	0.073	1.382	0.767–2.489	0.282
**Age group** (<65 vs. ≥65)	1.999	1.504–2.658	<0.001	2.080	1.564–2.767	<0.001
**Sex** (female vs. male)	1.155	0.840–1.588	0.376			
**Location** (distal vs. proximal)	1.091	0.814–1.461	0.562			
Histologic type* (differentiated vs. undifferentiated and others)	0.963	0.725–1.280	0.797			
**Lauren classification** (intestinal vs. diffuse and mixed)	1.095	0.825–1.455	0.530			
**pT category** (T1-2 vs. T3-4)	3.616	2.705–4.832	<0.001			
**Nodal status** (negative vs. positive)	3.335	2.488–4.471	<0.001			
**Stage**^†^ (I vs. II, III)	3.586	2.655–4.843	<0.001	2.371	1.445–3.890	0.001
**Lymphovascular invasion** (absent vs. present)	2.805	2.071–3.800	<0.001	1.058	0.662–1.691	0.813
**Perineural invasion** (absent vs. present)	3.243	2.430–4.328	<0.001	1.658	1.060–2.593	0.027
**EBV status** (negative vs. positive)	1.032	0.588–1.812	0.913			
**MSI status** (MSI vs. MSS)	1.306	0.743–2.293	0.354			
**HER2 amplification** (negative vs. positive)	1.640	0.934–2.881	0.085			

*Differentiated: well-differentiated, moderately differentiated adenocarcinoma; undifferentiated: poorly differentiated, signet ring cell carcinoma; others: papillary, mucinous, adenosquamous, hepatoid, gastric carcinoma with lymphoid stroma, adenocarcinoma with choriocarcinomatous differentiation

^†^AJCC 8^th^ edition.

Abbreviations: HR, hazard ratio; 95% CI, 95% confidence interval.

**Fig 3:**
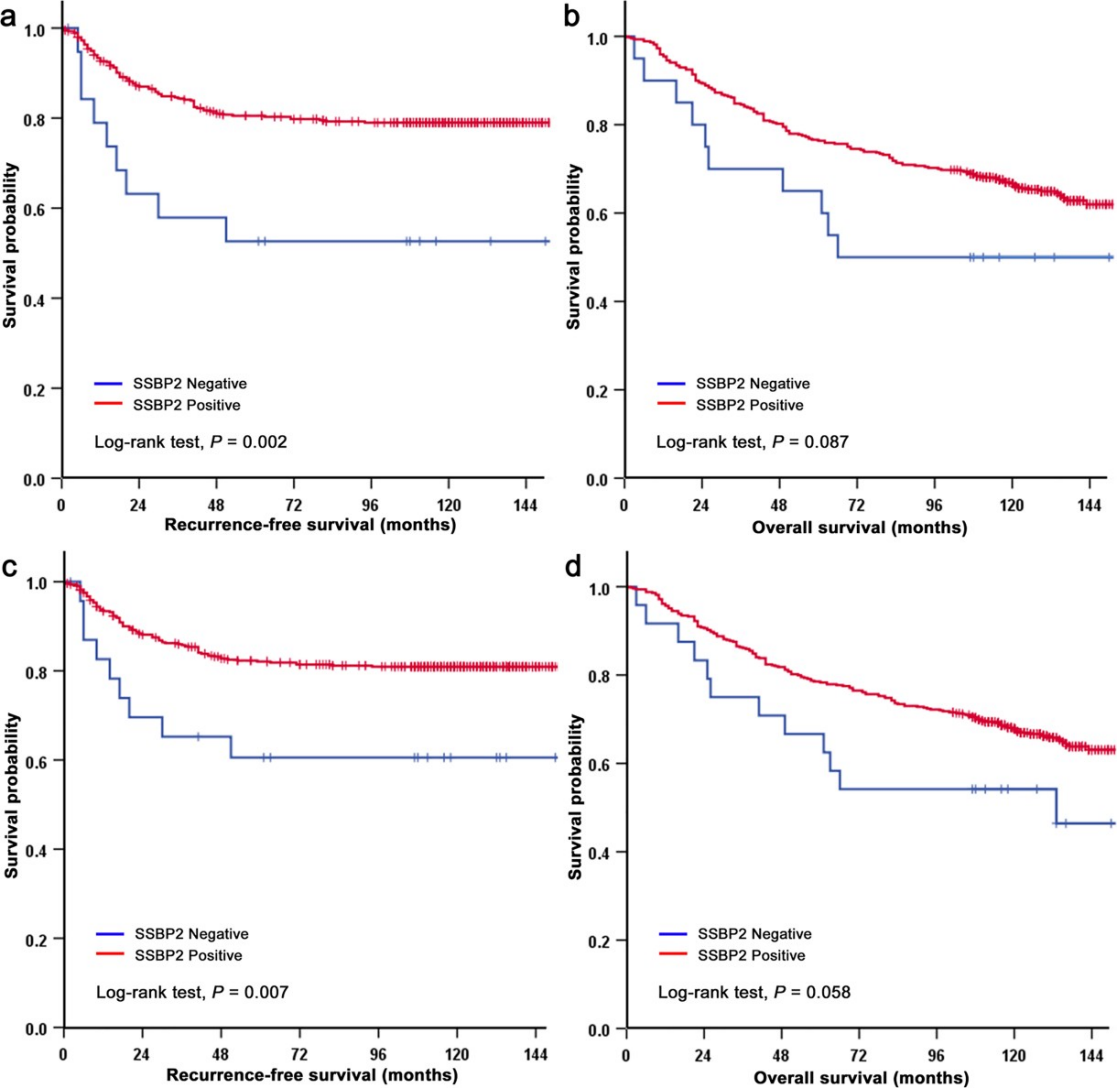
Kaplan-Meier analysis of SSBP2 in microsatellite stable (MSS) and EBV negative group (a and b), and in HER2 negative group (c and d). Recurrence-free survival (RFS) was significantly worse in patients with loss of SSBP2 expression compared to those with positive expression in MSS and EBV negative (a) and HER2 negative (c) groups. Overall survival (OS) was worse in patients with loss of SSBP2 expression compared to those with SSBP2 positive expression in MSS and EBV negative (b) and HER2 negative (d) groups.

In the MSS and EBV negative gastric cancer group, Kaplan-Meier survival curves also showed that a loss of nuclear SSBP2 expression was associated with shorter RFS and OS (*P* = 0.002 and *P* = 0.087, respectively; Fig 3A and 3B). However, the loss of SSBP2 expression lost statistical significance in the multivariate analysis (S3 Table). Of the cases with MSI, there was no recurrence during the follow-up period and SSBP2 expression had no prognostic implication for OS in MSI groups. In the HER2 negative group, patients with loss of SSBP2 expression also displayed shorter RFS and OS (*P* = 0.007 and *P* = 0.058, respectively; Fig 3C and 3D). Once again, the loss of SSBP2 expression lost statistical significance during the multivariate analyses (S4 Table).

We also divided into subgroups receiving fluoropyrimidine-based chemotherapy and groups receiving platinum-based chemotherapy and fluoropyrimidine plus platinum chemotherapy and analyzed the difference in the clinical outcome of each group according to the loss of SSBP2 expression. SSBP2 expression had no prognostic implication for RFS and OS in fluoropyrimidine-based chemotherapy group (*P* = 0.818 and *P* = 0.790, respectively). However, loss of SSBP2 expression was significantly associated shorter OS (*P* = 0.024) in platinum-based chemotherapy (with or without fluoropyrimidine) group, and the differences were not statistically significant in RFS (*P* = 0.114).

## Discussion

This study showed that loss of SSBP2 expression is: significantly associated with stage progression and poor prognosis (1), associated with poor prognosis in MSS/EBV negative and HER2 negative subtypes (2), and not correlated with molecular characteristics (3).

The effect of SSBP2 on the carcinogenesis and progression of malignant tumors is not yet clear. One of the known functions of SSBP2 is to bind LDB1 and regulate LDB1 activity. LDB1 regulates the differentiation of luminal cells in the gastrointestinal tract [17] and cardiomyocytes [18] and is also involved in cancer progression [11, 19]. Several studies on the promoter methylation of *SSBP2* in tumors have shown that it is one of the genes downregulated by methylation [13–15, 20, 21]. Promoter hypermethylation of SSBP2 was observed in several solid tumors, including gall bladder cancer, esophageal squamous cell carcinoma, prostate cancer, ovarian cancer, and hepatocellular carcinoma [13–15, 20, 22]. SSBP2 functioned as a tumor suppressor in gall bladder cancer, esophageal squamous cell carcinoma, and prostate cancer. Jun-Wei *et al*. [15] reported that *SSBP2* promoter hypermethylation was found in 61.4% of prostate cancer cases, whereas none of the benign prostatic hyperplasia cases showed hypermethylation. They also showed that SSBP2 expression, by immunohistochemistry, was significantly downregulated in most primary prostate cancer cases compared to normal prostatic tissues.

The prognostic impact of SSBP2 expression in gastric cancer, determined by immunohistochemistry, has not been reported. In this study, we investigated the expression of SSBP2 and its prognostic role in gastric adenocarcinoma. Focal or diffuse patterns of nuclear SSBP2 expression were observed in most cases (95.5%), with only 4.5% of all cases showing complete negativity. Complete loss of nuclear expression of SSBP2 was correlated with a high pT category and lymph node metastasis, suggesting an association with progression of the gastric adenocarcinoma. Loss of expression of SSBP2 was also associated with shorter RFS and OS.

We attempted to divide 539 adenocarcinoma cases according to the TCGA molecular classification. The immunohistochemistry for mismatch repair proteins and EBER-ISH was performed to identify the MSI and EBV subtype, while GS and CIN subtypes were not distinguished. The EBV subtype was 6.5%, and MSI subtype was 8.2% in our study. Results from the TCGA data indicated that the proportion of EBV and MSI groups was relatively small in our cohort (9% and 22% in TCGA, respectively) [5]. According to recent reports of subtype prognosis in the TCGA cohort, EBV subtypes have the best prognosis [23], although our study showed no significant difference. The number of positive HER2 positive cases in our study was smaller (4.8%). as compared to other studies (from 6.0 to 29.5%) [24]. HER2 positive cases had shorter OS than negative cases, but this was not statistically significant. Although all HER2 positive cases showed SSBP2 expression, there was no significant association between SSBP2 expression with EBV, MSI, and HER amplification status.

There were several limitations to our study. This was a retrospective study conducted on 539 cases. Cases of EBV, MSI, and HER2 amplification subtype were too few to give significant results. Therefore, larger-scale studies are needed to confirm the association between molecular characteristics and SSBP2 expression and the prognostic significance of SSBP2 for each molecular characteristic. Also, we evaluated the expression of SSBP2 by immunohistochemistry only, and could not explain the underlying mechanism. The association of molecular alterations according to the molecular subtypes of gastric cancer, such as MLH1 hypermethylation, and promoter methylation of SSBP2 is not yet known. Further studies are needed to confirm the association between molecular subtypes and SSBP2, and these studies will also help these studies will help to understand the role of SSBP2 and its effect on carcinogenesis and cancer progression in gastric cancer.

In conclusion, we investigated the pattern of SSBP2 expression through immunohistochemistry, as well as its clinicopathological significance in patients with gastric adenocarcinoma. Loss of SSBP2 expression was a poor prognostic factor associated with stage progression and recurrence. We also reported that SSBP2 expression did not show significant differences according to molecular characteristics, including TCGA molecular subtype and HER2 status.

## Supporting information

S1 Table Clinicopathological characteristics of gastric adenocarcinoma patients (n = 539). (DOCX)

S2 Table Summary of molecular characteristics of gastric adenocarcinoma patients (n = 539). (PDF)

S3 Table The univariate and multivariate Cox regression analyses for recurrence-free survival (RFS) and overall survival (OS) in genomic stability and chromosomal instability cases (microsatellite stable and EBV negative) (n = 460). (PDF)

S4 Table The univariate and multivariate Cox regression analyses for recurrence-free survival (RFS) and overall survival (OS) in HER2 negative cases (n = 513). (PDF)
