# Supplementary material for: The loss of nuclear expression of single-stranded DNA binding protein 2 of gastric adenocarcinoma and its prognostic role: Analysis of molecular subtype
Source: PLoS One. 2020 Aug 3;15(8):e0236896. doi: 10.1371/journal.pone.0236896 (PMC7398516; doi:10.1371/journal.pone.0236896)
Supplement: S1 Table — (DOCX) [file pone.0236896.s001.docx]

**Supplementary Table 1.** Clinicopathological characteristics of gastric adenocarcinoma patients (n = 539)

| Characteristics | Case No. (%) |
| --- | --- |
| Age, median (range, year) | 62 (25-90) |
| Sex |  |
| Male | 377 (69.9%) |
| Female | 162 (30.1%) |
| Location (center of tumor) |  |
| Cardia | 15 (2.8%) |
| Fundus | 2 (0.4%) |
| Body | 179 (33.2%) |
| Angle | 24 (4.5%) |
| Antrum | 311 (57.7%) |
| Pylorus | 8 (1.5%) |
| Gross type of EGC |  |
| EGC type I | 15 (5.1%) |
| EGC type IIa | 29 (9.8%) |
| EGC type IIb | 38 (12.8%) |
| EGC type IIc | 162 (54.5%) |
| EGC type III | 14 (4.7%) |
| Mixed | 39 (13.1%) |
| Borrmann type of AGC |  |
| Borrmann type 1 | 5 (2.1%) |
| Borrmann type 2 | 51 (21.1%) |
| Borrmann type 3 | 156 (64.5%) |
| Borrmann type 4 | 30 (12.4%) |
| Histologic type (by WHO classification, 2010) |  |
| Tubular adenocarcinoma, well differentiated | 72 (13.4%) |
| Tubular adenocarcinoma, moderately differentiated | 170 (31.5%) |
| Tubular adenocarcinoma, poorly differentiated | 109 (20.2%) |
| Papillary adenocarcinoma | 2 (0.4%) |
| Mucinous adenocarcinoma | 17 (3.2%) |
| Poorly cohesive carcinoma (including signet ring cell carcinoma) | 83 (15.4%) |
| Gastric carcinoma with lymphoid stroma | 19 (3.5%) |
| Adenosquamous cell carcinoma | 1 (0.2%) |
| Hepatoid adenocarcinoma | 1 (0.2%) |
| Mixed adenocarcinoma | 65 (12.1%) |
| Lauren classification |  |
| Intestinal | 267 (49.5%) |
| Diffuse | 142 (26.3%) |
| Mixed | 130 (24.1%) |
| Lymphovascular invasion |  |
| Present | 265 (49.2%) |
| Not identified | 274 (50.8%) |
| Perineural invasion |  |
| Present | 199 (36.9%) |
| Not identified | 340 (63.1%) |
| pT category |  |
| 1a | 181 (33.6%) |
| 1b | 116 (21.5%) |
| 2 | 47 (8.7%) |
| 3 | 102 (18.9%) |
| 4a | 86 (16.0%) |
| 4b | 7 (1.3%) |
| pN category |  |
| 0 | 322 (59.7%) |
| 1 | 57 (10.6%) |
| 2 | 62 (11.5%) |
| 3a | 47 (8.7%) |
| 3b | 51 (9.5%) |
| Stage^†^ |  |
| IA | 265 (49.2%) |
| IB | 42 (7.8%) |
| IIA | 53 (9.8%) |
| IIB | 33 (6.1%) |
| IIIA | 50 (9.3%) |
| IIIB | 45 (8.3%) |
| IIIC | 51 (9.5%) |
| Treatment |  |
| Surgery | 302 (56.0%) |
| Surgery + fluoropyrimidine-based chemotherapy | 129 (23.9%) |
| Surgery + platinum-based chemotherapy | 6 (1.1%) |
| Surgery + fluoropyrimidine plus platinum chemotherapy | 102 (18.9%) |

^†^AJCC 8^th^ edition

Abbreviations: AGC, advanced gastric cancers; EGC, early-stage gastric cancers
