# Supplementary material for: The loss of nuclear expression of single-stranded DNA binding protein 2 of gastric adenocarcinoma and its prognostic role: Analysis of molecular subtype
Source: PLoS One. 2020 Aug 3;15(8):e0236896. doi: 10.1371/journal.pone.0236896 (PMC7398516; doi:10.1371/journal.pone.0236896)
Supplement: S2 Table — (PDF) [file pone.0236896.s002.pdf]

S2 Table. Summary of molecular characteristics of gastric adenocarcinoma patients (n = 539)

EBV and MSI status	Case No. (%)
EBV positive	35 (6.5%)
MSI	44 (8.2%)
EBV negative and MSS	460 (85.3%)
**HER2 status**	**Case No. (%)**
Amplification	26 (4.8%)
No amplification	513 (95.2%)

Abbreviations: EBV, Epstein-Barr virus; MSI, Microsatellite instability; MSS, Microsatellite stable; HER2, human epidermal growth factor receptor 2
