# Supplementary material for: The loss of nuclear expression of single-stranded DNA binding protein 2 of gastric adenocarcinoma and its prognostic role: Analysis of molecular subtype
Source: PLoS One. 2020 Aug 3;15(8):e0236896. doi: 10.1371/journal.pone.0236896 (PMC7398516; doi:10.1371/journal.pone.0236896)
Supplement: S3 Table — (PDF) [file pone.0236896.s003.pdf]

S3 Table. The univariate and multivariate Cox regression analyses for recurrence-free survival (RFS) and overall survival (OS) in genomic stability and chromosomal instability cases (microsatellite stable and EBV negative) (n=460)

Recurrence-free survival (RFS)						
Variables	Univariate analysis			Multivariate analysis		
	HR	95% CI	*P*-values	HR	95% CI	*P*-values
SSBP2 expression (positive vs. negative)	2.833	1.427-5.626	0.002	1.762	0.881-3.525	0.109
Age group (<65 vs. ≥65)	1.082	0.717-1.631	0.708			
Sex (female vs. male)	1.001	0.652-1.539	0.995			
Location (distal vs. proximal)	0.976	0.648-1.472	0.910			
Histologic type* (differentiated vs. undifferentiated and others)	1.941	1.258-2.995	0.003			
Lauren classification (intestinal vs. diffuse and mixed)	2.036	1.330-3.116	0.001	1.370	0.887-2.116	0.155
pT category (T1-2 vs. T3-4)	11.032	6.532-18.634	<0.001			
Nodal status (negative vs. positive)	10.567	6.091-18.334	<0.001			
Stage† (I vs. II, III)	15.204	7.897-29.271	<0.001	6.175	2.367-16.107	<0.001
Lymphovascular invasion (absent vs. present)	11.465	5.957-22.066	<0.001	2.085	0.844-5.153	0.111
Perineural invasion (absent vs. present)	7.889	4.865-12.794	<0.001	1.444	0.792-2.632	0.230

Overall survival (OS)						
Variables	Univariate analysis			Multivariate analysis		
	HR	95% CI	*P*-values	HR	95% CI	*P*-values
SSBP2 expression (positive vs. negative)	1.735	0.915-3.289	0.088	0.764	0.400-1.458	0.414
Age group (<65 vs. ≥65)	2.110	1.553-2.865	<0.001	2.051	1.509-2.789	<0.001
Sex (female vs. male)	1.276	0.901-1.806	0.170			
Location (distal vs. proximal)	1.058	0.771-1.452	0.726			
Histologic type* (differentiated vs. undifferentiated and others)	1.018	0.749-1.383	0.911			
Lauren classification (intestinal vs. diffuse and mixed)	1.088	0.801-1.477	0.590			

pT category (T1-2 vs. T3-4)	3.865	2.823-5.292	<0.001			
Nodal status (negative vs. positive)	3.626	2.634-4.993	<0.001			
Stage[†] (I vs. II, III)	3.908	2.814-5.427	<0.001	2.814	1.572-5.038	<0.001
Lymphovascular invasion (absent vs. present)	2.935	2.111-4.082	<0.001	1.024	0.600-1.746	0.931
Perineural invasion (absent vs. present)	3.277	2.400-4.473	<0.001	1.437	0.893-2.312	0.135

[*]Differentiated: well-differentiated, moderately differentiated adenocarcinoma; undifferentiated: poorly differentiated, signet ring cell carcinoma; others: papillary, mucinous, adenosquamous, hepatoid, gastric carcinoma with lymphoid stroma, adenocarcinoma with choriocarcinomatous differentiation; [†]AJCC 8th edition

Abbreviations: HR, hazard ratio; 95% CI, 95% confidence interval
