# Supplementary material for: The loss of nuclear expression of single-stranded DNA binding protein 2 of gastric adenocarcinoma and its prognostic role: Analysis of molecular subtype
Source: PLoS One. 2020 Aug 3;15(8):e0236896. doi: 10.1371/journal.pone.0236896 (PMC7398516; doi:10.1371/journal.pone.0236896)
Supplement: S4 Table — (PDF) [file pone.0236896.s004.pdf]

S4 Table. The univariate and multivariate Cox regression analyses for recurrence-free survival (RFS) and overall survival (OS) in HER2 negative cases (n=513)

Recurrence-free survival (RFS)						
Variables	Univariate analysis			Multivariate analysis		
	HR	95% CI	*P*-values	HR	95% CI	*P*-values
SSBP2 expression (positive vs. negative)	2.468	1.244-4.899	0.008	1.685	0.846-3.359	0.138
Age group (<65 vs. ≥65)	1.005	0.669-1.511	0.980			
Sex (female vs. male)	0.907	0.585-1.404	0.660			
Location (distal vs. proximal)	0.864	0.577-1.293	0.478			
Histologic type* (differentiated vs. undifferentiated and others)	2.002	1.293-3.101	0.002			
Lauren classification (intestinal vs. diffuse and mixed)	2.169	1.418-3.317	<0.001	1.431	0.929-2.202	0.104
pT category (T1-2 vs. T3-4)	11.080	6.565-18.700	<0.001			
Nodal status (negative vs. positive)	10.802	6.231-18.728	<0.001			
Stage† (I vs. II, III)	13.764	7.348-25.780	<0.001	5.428	2.269-12.984	<0.001
Lymphovascular invasion (absent vs. present)	10.242	5.470-19.180	<0.001	2.080	0.889-4.866	0.091
Perineural invasion (absent vs. present)	7.990	4.930-12.947	<0.001	1.564	0.839-2.917	0.159

Overall survival (OS)						
Variables	Univariate analysis			Multivariate analysis		
	HR	95% CI	*P*-values	HR	95% CI	*P*-values
SSBP2 expression (positive vs. negative)	1.748	0.973-3.141	0.058	1.440	0.798-2.599	0.226
Age group (<65 vs. ≥65)	1.940	1.445-2.605	<0.001	2.024	1.506-2.720	<0.001
Sex (female vs. male)	0.848	0.609-1.181	0.328			
Location (distal vs. proximal)	0.908	0.670-1.229	0.531			
Histologic type* (differentiated vs. undifferentiated and others)	0.952	0.709-1.279	0.745			
Lauren classification (intestinal vs. diffuse and mixed)	1.090	0.812-1.463	0.566			
pT category (T1-2 vs. T3-4)	3.541	2.623-4.781	<0.001			

Nodal status (negative vs. positive)	3.293	2.431-4.460	<0.001			
Stage[†] (I vs. II, III)	3.397	2.493-4.630	<0.001	2.135	1.279-3.564	0.004
Lymphovascular invasion (absent vs. present)	2.773	2.025-3.796	<0.001	1.071	0.653-1.757	0.785
Perineural invasion (absent vs. present)	3.198	2.371-4.314	<0.001	1.748	1.090-2.804	0.021

[*]Differentiated: well-differentiated, moderately differentiated adenocarcinoma; undifferentiated: poorly differentiated, signet ring cell carcinoma; others: papillary, mucinous, adenosquamous, hepatoid, gastric carcinoma with lymphoid stroma, adenocarcinoma with choriocarcinomatous differentiation; [†]AJCC 8th edition

Abbreviations: HR, hazard ratio; 95% CI, 95% confidence interval
